# Treatment of a Non-syndromic Carotid Body Paraganglioma Using Fast Neutron Radiotherapy: A Case Report and Review of Literature

**DOI:** 10.7759/cureus.44673

**Published:** 2023-09-04

**Authors:** Siddharth Ramanathan, Bailey A Loving, James Fontanesi

**Affiliations:** 1 Radiation Oncology, Oakland University William Beaumont School of Medicine, Rochester Hills, USA; 2 Radiation Oncology, Corewell Health, Royal Oak, USA

**Keywords:** pre-treatment embolization, radiation therapy, neutron therapy, glomus tumor, carotid body paraganglioma

## Abstract

Non-syndromic carotid body paragangliomas (CBPs) are the most common head and neck CBPs. Malignant transformation or symptomatic presentation is rare, but patients may occasionally endorse tinnitus, cranial nerve (CN) deficits, and ear pulsations. Historically, treatment of CBP was primarily through surgical intervention, which predisposed patients to CN deficits and significant blood loss due to the neurovascular structures in close proximity to these tumors. More recently, the utilization of pre-treatment embolization and radiotherapy has allowed for the reduction in treatment morbidity. Stereotactic radiosurgery (SRS) and external beam radiotherapy (EBRT) have been investigated as alternatives to traditional surgical intervention, with a documented reduction in the incidence of postoperative morbidity. While several retrospective studies and meta-analyses compare outcomes following surgical and traditional radiotherapeutic interventions, currently no literature exists regarding the potential utility of fast neutron therapy in treating this disease. In this case report, we highlight a patient with a non-syndromic CBP treated with pre-treatment embolization and fast neutron therapy, review the post-treatment course, and present a review of the extant literature on the subject.

## Introduction

Non-syndromic carotid body paragangliomas (CBPs), also known as glomus tumors, are the most commonly occurring subtype of head and neck CBPs [1]. These neoplasms often present as a painless neck mass in middle-aged adults, but can also cause ear pain, pulsation, tinnitus, and cranial nerve (CN) palsies [1]. CBPs are usually benign, with only 1% to 5% of cases exhibiting malignant traits. The incidence of lymph node or distant metastasis is rare, occurring in less than 5% of cases [1-2]. Tumor size, endocrine activity, and degree of vascular involvement are critical factors that influence the staging and symptomatology of patients with CBPs. Patients with larger tumors may experience neurological deficits relating to CNs X-XII. Secretory CBP, which makes up a small proportion of all CBPs, can increase plasma catecholamine levels resulting in sporadic hypertension, flushing, and palpitations, among other catecholamine-related symptoms. Parasympathetic predominance is even rarer and usually endocrinologically silent, posing a lower risk of malignant transformation compared to sympathetic CBPs. While a genetic component has been shown to play a significant role in the development of CBP, this case study focuses only on sporadic CBP [2].

The management of patients with CBPs is determined by several factors, including patient age, tumor size, tumor location, and physician preference. Although routine follow-up is recommended for patients with non-syndromic asymptomatic CBPS, intervention may be indicated for patients with CN deficits, symptoms of mass effect, or tumors with greater degrees of vascular involvement [2-3]. Surgical resection remains the primary treatment option for CBPs, and it poses up to a 30% to 40% risk of postoperative morbidity and mortality due to the proximity of these tumors to CN and the potential for massive blood loss due to vascular involvement [3]. Preoperative endovascular embolization can be performed to mitigate the potential for blood loss in these hypervascular tumors. Although current data on newer polymers has shown a reduction in these post-treatment morbidities, endovascular embolization still increases the risk for other major complications including CN palsies and toxicity [4-5]. Therefore, stereotactic radiosurgery (SRS) has emerged as a promising nonoperative treatment for patients who have relatively large CBPs with involvement of the lower CNs or patients who are not suitable surgical candidates. SRS is also commonly used as an adjuvant treatment following incomplete surgical resection to slow tumor growth and potentially improve outcomes [5].

Nevertheless, reports estimate the incidence of CN dysfunction at 30% for patients treated with SRS [6]. External beam radiotherapy (EBRT) is an alternative to SRS, which attenuates the risk for CN dysfunction through dose fractionation. EBRT is an effective treatment option with excellent local control [7]. However, EBRT requires a longer course of radiation treatment and increases the radiation dose administered to native tissues, adding additional burden to the patient [6-7]. Ultimately, the choice of treatment depends on individual circumstances and should be made after a careful multidisciplinary review including a neurosurgeon, neuroradiologist, and radiation oncologist.

Neutron therapy may also serve as an adjunctive therapy for large, unresectable CBPs. This treatment modality takes advantage of the high linear energy transfer (LET) of neutron beams to cause DNA breaks in rapidly dividing tumor cells. Unlike radiation modalities with lower LETs, the higher LET of neutron therapy causes double-stranded breaks in tumor DNA, effectively killing the tumor cell. Compared to radiotherapy treatments utilizing charged particles such as proton therapy, neutron therapy itself is not affected by the electrostatic reactions from protons and electrons. However, fast neutron therapy is unable to utilize the phenomenon of Bragg peaks at maximal radiation dosage depth and also does not create free radicals, making neutron therapy less accurate than other radiation modalities. However, for tumors of neuronal origin specifically, neutron therapy may possess unique benefits due to the creation of hydrogen protons through reactions with tumors containing high contents of fatty acids, such as sphingomyelin [8].

This case highlights one such instance where neutron therapy was felt to be an appropriate treatment option due to the patient’s age and surgical risk factors in conjunction with the theoretical efficacy of fast neutron therapy in treating CBPs due to their neuroendocrine origin. In this case, we highlight the clinical course of a 43-year-old female who underwent embolization followed by neutron therapy for the treatment of a right-sided CBP. This case presents a unique treatment for a rare malignancy, achieving long-term local control without appreciable radiation toxicity.

## Case presentation

A 43-year-old female with a medical history significant for hypertension and chronic kidney disease presented in 1995 with a chief complaint of an isolated right-sided neck mass. The patient had no other contributory medical or family history. A contrast-enhanced CT scan revealed a 3 x 2.5 cm mass located medial to the right common carotid artery, with early-phase vascular filling. A biopsy was performed to establish the diagnosis, and the results were consistent with the diagnosis of a Shamblin grade III CBP. The patient underwent vascular embolization followed by neutron radiation therapy and tolerated the treatment without notable side effects. She was followed up routinely by her treating physician and received regular surveillance CT imaging, without any signs of tumor recurrence. In 2007, the decision was made to continue with routine clinical follow-up alone.

In 2018, 23 years after her initial treatment, the patient presented with new complaints of dysphagia and regurgitation. MRI showed a mediastinal mass measuring 0.95 x 0.76 x 0.85 cm located posterior to the left lower lobe of the thyroid, posterolateral to the trachea, and posteromedial to the carotid artery. Evaluation of the right carotid space of the prior radiation bed did not reveal any changes from prior imaging in 2007. After consulting with the patient and presenting the case to a multidisciplinary tumor board, it was decided to monitor the mediastinal mass with routine ultrasound.

**Figure 1:**
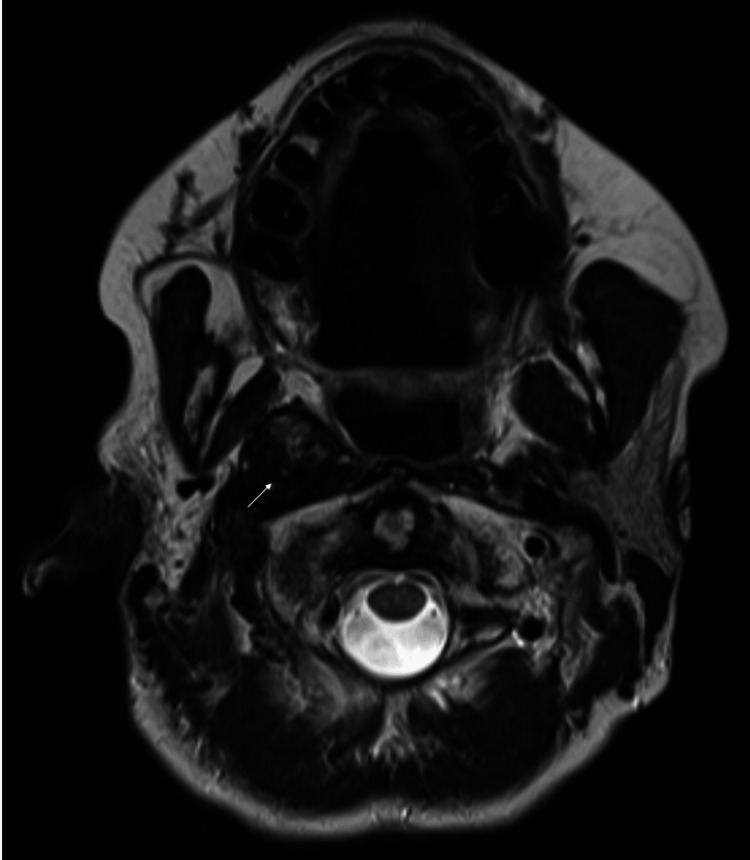
T2-weighted MRI of prior radiation bed (arrow) in December 2021 demonstrating no increase in size from the previous scan

**Figure 2:**
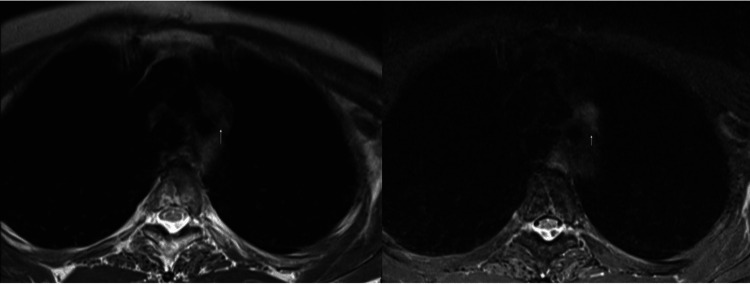
T2-weighted MRI (left) and MRI STIR sequence (right) on December 2021 demonstrating a new mediastinal mass (arrows)

**Figure 3:**
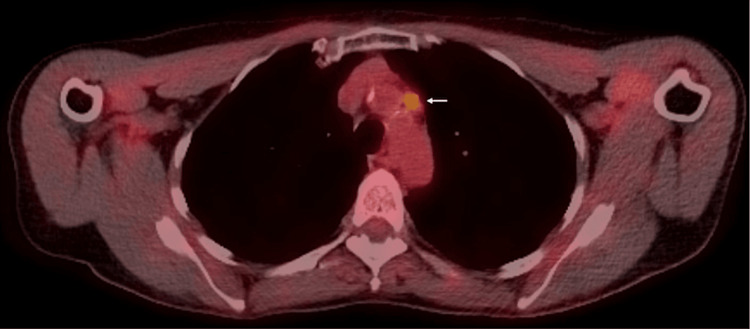
PET/CT completed in February 2022 demonstrating an increased FDG uptake in mediastinal mass (arrow)

In December 2021, the patient's difficulty swallowing had worsened since the previous follow-up visit, warranting a complete investigative workup. An MRI of her neck revealed a 3.1 x 1.7 x 4.4 cm mass medial to the right carotid body extending from the skull base superiorly to approximately C2-C3 inferiorly (Figure 1) and a 1.3 x 1.1 x 1.1 cm mediastinal nodule posterior to the left thyroid lobe (Figure 2). These masses were heterogeneously enhancing, with mixed, predominantly hyperintense, T2 and short tau inversion sequence (STIR) signal intensities and low T1 signal intensity. Combined PET/CT results showed no significant (18F)-fluorodeoxyglucose (FDG) radiotracer uptake in either carotid body and no change in the size of the right carotid body mass. However, there was increased FDG uptake in a mediastinal focus posteroinferior to the left lobe of the thyroid with a maximum standardized uptake value of 6.1 and a maximum dimension of 1.4 cm (Figure 3).

Due to the non-diagnostic findings and concern for a metastatic lymph node or new primary mediastinal malignancy, a biopsy was scheduled with thoracic surgery. The biopsy results in June 2022 demonstrated tumor cells in a nesting pattern with positive staining for chromogranin, synaptophysin, S100, and SOX-10, supporting the diagnosis of CBP in the mediastinal mass with no signs of malignancy in the previously treated tumor bed. After discussing the case with the multidisciplinary tumor board and with the patient, the decision was made to forgo any invasive procedures in accordance with the patient's wishes.

## Discussion

Surgical resection is the preferred treatment for CBPs, but it may not be feasible in all cases. In this case, the patient was not a surgical candidate due to the high risk of morbidity associated with surgery, and, therefore, vascular embolization followed by neutron therapy was used as an alternative treatment modality. The combination of vascular embolization and radiation therapy has been reported to be an effective treatment option for patients with CBPs who are not surgical candidates [9]. In this case, the patient tolerated the treatment well, and surveillance CT scans showed no evidence of tumor recurrence on long-term follow-up.

The management of CBP is a complex and evolving topic that requires careful consideration of various factors. While recent studies have validated the efficacy of irradiation over surgical resection in treating CBP, especially Shamblin type II or III CBPs, the management of this condition remains intrinsically linked to the location and behavior of the tumor itself [10-11]. More aggressive tumors, secretory tumors, and tumors with complex association with the adventitial layer of the vessel often require surgical resection. Significant controversy exists regarding factors determining the metastatic risk of CBPs. However, several studies have identified tumor size, the presence of biochemical enzyme mutations, and secretory tumors as potential risk factors for metastatic presentation [12].

Following diagnostic confirmation of CBP either through tumor biopsy or angiography, patients deemed appropriate candidates for surgical or radiotherapeutic interventions may require further treatment. A large meta-analysis demonstrated high rates of local control in patients treated with surgical resection (>94%) [11]. However, this study also uncovered an increasing frequency of CN deficits with higher Shamblin classifications (29% CN deficit in patients with Shamblin type III CBP) and higher rates of serious adverse events with higher Shamblin classifications (10% in patients with Shamblin type III CBP) [11]. A large meta-analysis of patients with head and neck paragangliomas treated with SRS found a >94% rate of local control with low rates of hearing loss (<5%), tinnitus (<2%), and CN deficit (<2%) [9]. In a meta-analysis of patients with CBPs treated with EBRT, results demonstrated high rates of local control (93%). However, this study did not assess rates of serious adverse events or CN palsies [13].

Despite advances in treatment, the rate of metastasis post-treatment remains poorly understood. A 45-year review of patients with CBP treated with radiotherapy found an extremely low rate of tumor recurrence (3.2%) or tumor metastasis (<1%) [7]. However, there was no analysis of the specific Shamblin type of the patients in this series. Contrastingly, there is virtually no literature on the treatment and long-term metastatic risk of patients with CBP treated with neutron therapy.

This case report describes a patient who was treated with embolization and neutron beam therapy for a right-sided CBP, only to develop a metastatic mediastinal CBP over two decades later. While we were unable to highlight specific dosimetric data, we still believe this report adds context to the existing literature on the management of CBPs. Due to the patient's age and the significant risk factors for morbidity, surgical excision was not felt to be a feasible treatment option. The decision to treat with neutron beam therapy was made after considering the patient's emphasis on maintaining the quality of life and the documented efficacy of fast neutron beams in the treatment of tumors of neuronal origin. Following treatment, this patient experienced a symptom-free interval of over 20 years, but despite the long-term control, the tumor subsequently recurred. The adverse effects of surgical intervention and EBRT in managing high-grade CBPs have been well documented [7-13]. However, in this patient, no significant long-term adverse effects were observed following treatment with neutron beam therapy. This is the first report of its kind in the literature which highlights the need for further investigation into the efficacy of neutron beam therapy in the treatment of CBP, as well as the long-term treatment outcomes in this patient population.

Overall, this case report underscores the importance of carefully assessing each patient's unique circumstances when managing CBPs. Treatment decisions should be based on a thorough understanding of the risk factors for metastatic presentation, the available treatment options, and the potential long-term sequelae. Further research is needed to better understand the optimal management strategies for patients with CBP, particularly those who have been treated with novel therapies such as neutron beam therapy.

## Conclusions

CBPs are rare tumors that can be challenging to treat. Although neutron therapy is no longer a widely used treatment modality, vascular embolization followed by neutron therapy proved to be a viable treatment option for this patient. In this case, the patient remained disease-free for 23 years after treatment.
